# miR-34a-5p-mediated inhibition on the KRT5/DSP axis blunts cancer stem cell properties and cisplatin resistance in non-small cell lung cancer

**DOI:** 10.1007/s12672-026-05424-z

**Published:** 2026-06-23

**Authors:** Yelin Song, Zuowei Li

**Affiliations:** 1 General Practice Department, Qingdao Hiser Hospital Affiliated of Qingdao University (Qingdao Traditional Chinese Medicine Hospital), Qingdao, 266034 Shandong China; 2 Shandong University of Traditional Chinese Medicine, Jinan, 250000 Shandong China; 3 Qingdao Medical College, Qingdao University, Qingdao, 266073 Shandong China; 4 Department of Neurology, Affiliated Hospital of Shandong University of Traditional Chinese Medicine, Jinan, 250000 Shandong China

**Keywords:** MicroRNA-34a-5p, NSCLC, KRT5, DSP, Cisplatin resistance, CSC

## Abstract

The survival rate of patients diagnosed with non-small cell lung cancer (NSCLC) are significantly influenced by drug resistance and metastasis with cancer stem cells (CSCs). MicroRNAs (miRNAs) impact on the progression of various human diseases, including different types of cancers. This research seeks to clarify how miR-34a-5p influences CSC characteristics and resistance to cisplatin (DDP) in NSCLC. Initially, the levels of expression for miR-34a-5p, KRT5, and DSP were analyzed in both NSCLC tissue samples and cell lines through RT-qPCR and western blot techniques. Subsequently, sphere formation assays, CCK-8 assays, and flow cytometry tests were carried out to evaluate CSCs and their resistance to DDP in NSCLC. The expression of genes related to stemness, such as SOX2, CD133, and ALDH, was assessed using western blotting methods. Functional experiments were also conducted to investigate the regulatory mechanisms linked to miR-34a-5p, KRT5, and DSP. Finally, the effect of miR-34a-5p on the CSC phenotype and resistance to DDP was validated using a xenograft mouse model. Results showed that, in NSCLC patients, miR-34a-5p was found to be decreased, while KRT5 and DSP expressions were elevated. By targeting the KRT5/DSP axis, miR-34a-5p was shown to suppress CSC properties and enhance DDP sensitivity in NSCLC cells. miR-34a-5p directly bound to and inhibited KRT5, resulting in the downregulation of DSP expression. Furthermore, the overexpression of miR-34a-5p in mice resulted in reduced tumor growth. Collectively, this study elucidates the regulatory role of miR-34a-5p on CSC properties, providing insights that may aid in overcoming drug resistance in NSCLC.

**Supplementary Information:**

The online version contains supplementary material available at https://doi.org/10.1007/s12672-026-05424-z.

## Introduction

Globally, lung cancer ranks as the second most prevalent malignancy, representing 11.4% of total cancer incidence. It is also the leading cause of cancer-associated mortality, responsible for 18% of all cancer deaths—approximately 1.8 million fatalities annually [1]. Among patient with lung cancer, approximately 85% of the cases are diagnosed as NSCLC [2]. Conventional doublet chemotherapy regimens containing platinum agents remain the gold standard of care for patients with NSCLC but unavailable to uproot metastasis or rather the disease-related deaths due to drug resistance [3]. CSCs are defined as neoplastic cells possessing stem cell-like phenotypes, and they are hypothesized to drive the development of resistance to antineoplastic interventions including chemotherapy, radiotherapy, and targeted therapies [2, 4]. In NSCLC, CD133 and ALDH1 are two markers widely used for the identification of CSCs [5]. Based on clinical and experimental evidences, existing chemotherapeutic regimens fail to target human CSCs—a cell population recognized as the primary driver of disease recurrence.[6]. This highlights the imperative for an actionable strategy to overcome chemoresistance induced by CSCs.

MicroRNAs (miRNAs) serve as crucial regulators of various cellular activities. They exert their regulatory effects by attaching to complementary sequences, usually found within the 3’UTR of target messenger RNAs (mRNAs). Thereby triggering mRNA cleavage or suppressing protein translation [7]. miRNAs implicates in inhibiting or promoting NSCLC development [8, 9], and have an emerging role in regulating CSCs as well [10, 11]. miR-34a could act as a tumor suppressor gene through restraining the drug resistance of NSCLC cells [12]. It has already been reported to negatively regulate tumorigenic properties of NSCLC cells and stem-like NSCLC cells [13], but there are still many issues to be investigated, for example, which downstream targets are regulated by miR-34a-5p for triggering CSC properties and CSC-induced chemoresistance.

Keratin 5 (KRT5) was previously appreciated as an immunomarker in distinguishing LSCC from LUAD [14]. KRT5 was increasingly expressed in LUAD tissues and inhibiting its expression contributed to preventing LUAD [15]. Desmoplakin (DSP) was found to be strongly expressed in LSCC cells [16]. However, emerging evidence indicates that its expression is significantly downregulated across multiple lung cancer cell line models [17]. Notably, miR-34b and miR-34c—two members of the miR-34 family—were found to upregulate desmoplakin (DSP) expression in lung adenocarcinoma (LUAD) cells [18].

Importantly, bioinformatics tools suggested differentially expressed KRT5 and DSP in lung cancer and the correlations between miR-34a-5p and KRT5/DSP expression. It is plausible to postulate that miR-34a-5p might modulate stemness-associated NSCLC cells and chemoresistance via the regulation of KRT5 and DSP.

## Materials and methods

### Clinical tissues

All clinical sample collection procedures in this study were conducted in accordance with the principles of the Declaration of Helsinki and were approved by the Ethics Committee. Prior to sample collection, each participant was informed of the research purpose, procedures, and potential risks, and provided written informed consent. Tumor tissues and adjacent normal tissues were collected from 48 patients who underwent surgical treatment at our hospital between 2017 and 2020. None of these patients had received chemotherapy, radiotherapy, or biological therapy. Tumor staging was performed according to the 6th edition of the AJCC TNM staging criteria. All tumor tissue samples were obtained through surgical resection or needle biopsy. After surgical resection, the pathologist promptly excised tumor tissue blocks approximately 0.5 cm in diameter under sterile conditions, while making every effort to collect paired adjacent normal tissues (located > 2 cm from the tumor margin). All pathological diagnoses were confirmed by two senior pathologists. And the clinicopathological characteristics of the patients were registered and presented in Table 1.

**Table 1:** The relevance of miR-34a-5p/KRT5 expression with the pathological features of NSCLC patients

Characteristic	Case(N)	miR-34a-5p expression		P Value	KRT5 mRNA expression		P Value
		High	Low		High	Low	
		(n = 24)	(n = 24)		(n = 26)	(n = 22)	
Age (years)							
≥ 55	28	15	13	0.558	14	14	0.493
< 55	20	9	11		12	8	
Gender							
Male	26	12	14	0.579	13	13	0.529
Female	22	12	10		13	9	
Pathological Type							
Squamous	20	11	9	0.799	9	11	0.547
Adenocarcinoma	12	6	6		7	5	
Large Cell Lung cancer	16	7	9		10	6	
Smoking status							
Non-smoker	14	6	8	0.471	5	9	0.214
Smoker	16	10	6		9	7	
Heavy smoker	18	8	10		12	6	
Lymphatic Metastasis							
No	27	17	10	0.042*	8	19	0.0001***
Yes	21	7	14		18	3	
TNM stage							
I/II	18	14	4	0.003**	5	13	0.005**
III/IV	30	10	20		21	9	

TNM, tumor lymph node metastasis; NSCLC, non-small cell lung cancer

### Animal and ethics statement

The 6-week-old female nude mice used in this study, with body weights of (20 ± 5) g, were purchased from Henan Scibase Biotechnology Co., Ltd. The laboratory animal qualification certificate number: No.410983241100623283; experimental unit usage license number: SYXK (Lu) 20220009. Tumor-bearing procedures were performed after one week of adaptive feeding in the animal facility. All animal experimental protocols in this study were approved by the Ethics committee of Shandong University of Traditional Chinese Medicine. According to the approved protocol, the maximum allowable tumor volume in this study should not exceed 1500 mm^3^. Throughout the experiment, researchers measured tumor size and animal body weight every two days. We confirm that the tumor burden of all experimental animals did not exceed the maximum limit set by the aforementioned ethics committee prior to euthanasia. Should any individual animal exhibit abnormalities during the experiment, humanitarian endpoint assessment will be immediately conducted according to the protocol, followed by premature euthanasia, with the relevant data excluded from final analysis.

After one week of adaptive feeding, the tumor-bearing experiment was conducted. According to the protocol provided by the manufacturer (RiboBio), the Agomir-miR-34a-5p transfection reagent was prepared, and 3 × 10^5 cells were seeded into 12-well plates with a transfection concentration of 50 nM. After transfection, the cells were subcutaneously injected into the right abdomen of the mice. Tumor growth was monitored every five days. At the end of the xenograft experiment, the mice were euthanized by cervical dislocation, and the tumors were excised and weighed. Paraffin sections of the xenografts were then prepared and analyzed by immunohistochemistry using an anti-Ki67 antibody (1:1000, #12202, Cell Signaling Technology, Denver, USA). For the xenotransplantation experiment, A549/DDP cells at specified concentrations (1 × 10⁶, 1 × 10^5^, or 1 × 10^4^ cells suspended in 100 μl DMEM; n = 6) were subcutaneously injected into mice. Five weeks post-injection, tumor volumes were measured and recorded.

### Cells and transfections

Human NSCLC cell lines (A549、H1299、SK-MES-1 and Calu-3) and human bronchial epithelial cell line (HBE) and Cisplatin-resistant lung adenocarcinoma cell line (A549/DDP) purchased from Procell (Wuhan, China). The Cisplatin-resistant human lung adenocarcinoma cell line (H1299/DDP) was purchased from Jinyuan Biotechnology (Shanghai, China). Two types of cells were maintained in RPMI-1640 under standard conditions (5% CO2, 37 °C). To maintain the cisplatin resistance of A549/DDP and H1299/DDP cells, cisplatin (MedChemExpress, Shanghai, China) was added to their culture medium at a concentration of 1 μg/mL. The cisplatin was dissolved in DMSO solution with a stock concentration of 5 mg/mL.

Seed 5 × 10^6 cells into 6-well plates. When cell confluence reaches 70% or higher, add the transfection complexes containing KRT5 short hairpin RNA (sh-KRT5, 2 μg), sh-DSP (2 μg) (Shanghai Sangon Biotech, Shanghai, China), miR-34a-5p mimic and inhibitor (100 nmol, RiboBio) to the cell culture dishes according to the Lipofectamine 2000 reagent (Invitrogen, California, USA) transfection protocol. Replace with fresh medium after 6 h of transfection, and incubate the cell culture plates in a 37 °C, 5% CO_2_ incubator for 48–72 h. Then perform transfection efficiency detection.

### CCK-8 assay for cisplatin resistance evaluation

Cell viability was assessed using the CCK-8 assay kit (Beyotime, China). Briefly, 8,000 cells per well were seeded in a 96-well plate and cultured overnight. Then, gradient concentrations of cisplatin (2, 4, 6, 8, 16, and 32 μM) were added, and the cells were incubated for an additional 24 h. Subsequently, 10 μL of CCK-8 reagent was added to each well and co-incubated for 2 h. The absorbance at 450 nm was measured using a microplate reader (Multiskan SkyHigh; Thermo Fisher Scientific). Cell viability at each drug concentration was calculated, and the half-maximal inhibitory concentration (IC50) was determined by nonlinear regression analysis.

### Sphere formation

Following dissociation, single-cell suspensions (5 × 10^3^ cells/well) were seeded into 6-well ultra-low attachment plates (Corning, NY, USA), and the plates were supplemented with DMEM/F12 medium containing 20 ng/mL fibroblast growth factor (FGF), 20 ng/mL epidermal growth factor (EGF), and 2% B27 supplement. After incubation for 2 ~ 3 weeks, The observation was captured at 100 × magnification under a microscope (Nikon, TI2-E). At least three random microscopic fields were selected, and the number of spheroids in each field was counted.

### Flow cytometry

According to the manufacturer's protocol, cell apoptosis was detected using the Annexin V-PE/7-AAD Apoptosis Detection Kit (YEASEN, Shanghai, China). In summary, Cells were stained with Annexin V-PE and 7-AAD, and the expression levels of various cell surface markers were detected using a flow cytometer (FC500, Beckman Coulter). The results were analyzed with FlowJo 10.8.1 software. PE fluorescence was detected at 565 nm wavelength, and 7-AAD fluorescence was detected at 488 nm wavelength. Three biological replicates were performed for each group.

Flow cytometry was also used to evaluate the expression levels of the CD133 surface marker in the transfected cells. Collect cells and prepare a cell suspension at a concentration of 3 × 10^5^ cells/mL. Add 1 μL of CD133 surface staining flow antibody (1:200; Cat. no. 11-1339-42; Invitrogen; Thermo Fisher Scientific), and incubate at room temperature protected from light for 30 min. After two washes with pre-cooled PBS, analyze the expression levels of cell surface markers using a flow cytometer (FC500, Beckman Coulter). Process the results using FlowJo 10.8.1 software.

### RT-qPCR

Total RNA was extracted from NSCLC cells or tissue samples via the Trizol reagent method (Takara Co., Ltd., Dalian, China), followed by quantification of RNA concentration and assessment of its purity. The cDNA templates were synthesized following the standard protocols provided by the TaKaRa miRNA cDNA Synthesis Kit (Cat.# 638315) and TaKaRa mRNA cDNA Synthesis Kit (Cat.# 6210A), and PCR amplification was performed using the TaKaRa RT-qPCR Kit. Expression levels of miRNAs and mRNAs were quantified and subsequently normalized to U6 small nuclear RNA (snRNA) and glyceraldehyde-3-phosphate dehydrogenase (GAPDH), respectively. Each RNA sample had three replicates and the results were analyzed using 2^−∆∆Ct^ method. Each experiment was repeated in triplicate. The synthesis of primers (Table 2) and PCR reaction were performed by RiboBio (Guangzhou, China).

**Table 2:** Primer sequences

Name of primer	Sequences
miR-34a-5p-F	TGGCAGTGTCTTAGCTGGTTGT
miR-34a-5p-R	GCGAGCACAGAATTAATACGAC
U6-F	CTCGCTTCGGCAGCACA
U6-R	AACGCTTCACGAATTTGCGT
KRT5-F	CCAAGGTTGATGCACTGATGG
KRT5-R	TGTCAGAGACATGCGTCTGC
DSP-F	TGAGCTGGCAAAGGCATCTA
DSP-R	TTCACCCTGGTTTCTGCCTC
SOX2-F	AACCAGCGCATGGACAGTTA
SOX2-R	CGAGCTGGTCATGGAGTTGT
PROM1-F	AGTCGGAAACTGGCAGATAGC
PROM1-R	GGTAGTGTTGTACTGGGCCAAT
ALDH1A1-F	GATCCCCGTGGCGTACTATG
ALDH1A1-R	TGGATCTTGTCAGCCCAACC
GAPDH-F	AGAAGGCTGGGGCTCATTTG
GAPDH-R	AGGGGCCATCCACAGTCTTC

F, forward; R, reverse

### Western blot

Cells or tissues were lysed using RIPA lysis buffer (Beyotime) supplemented with protease and phosphatase inhibitors (Beyotime) on ice. Protein concentrations were measured using the BCA protein assay kit (Beyotime). Equal amounts of protein were resolved with 1 × loading buffer on 7.5%–12.5% gels (Epizyme) following standard protocols, and transferred to PVDF membranes (Millipore) on ice. After blocking with 5% BSA, the membranes were incubated overnight at 4 °C with the following primary antibodies: GAPDH (1:3000; Proteintech; 60004-1-Ig), KPT5 (1:3000; PTG; 28506-1-AP), DSP (1:1000; Abcam; ab109445). The membranes were then washed with TBST (Beyotime) and incubated with corresponding HRP-conjugated secondary antibodies at room temperature for 2 h. The corresponding HRP-conjugated secondary antibody (Beyotime, 1:3000) was incubated at room temperature for 1 h, followed by chemiluminescence detection using the iBright imaging system (Thermo Fisher Scientific).

### Co-immunoprecipitation (Co-IP)

After extracting total proteins using cell lysis buffer, experiments were performed using a co-immunoprecipitation kit (Beyotime). Briefly, protein samples were incubated overnight at 4 °C with antibody- or normal IgG-conjugated magnetic beads. Following the binding of protein samples to corresponding antibodies, the suspensions were eluted and separated using magnetic beads for subsequent western blot analysis. The following antibodies were used: KRT5 (1:1000; Santa; sc-377431), DSP (1:1000; Santa; sc-365981).

### RNA pull-down

Three distinct types of miRNA sequences were meticulously designed and synthesized by Shanghai GenePharma Co., Ltd., located in Shanghai, China. These sequences were labeled with biotin for experimental clarity: the wild type miR-34a-5p, designated as Bio-miR-34a-5p-WT, the mutant variant of miR-34a-5p, known as Bio-miR-34a-5p-MUT—which has undergone mutation in the portion that is complementary to the KRT5 gene—and a negative control, which is a random miRNA that does not complement KRT5, referred to as Bio-NC. The cells were seeded in culture dishes at a density of 3 × 10^5 cells per well. When the cells reached 60–70% confluence, chemically synthesized target microRNAs (miRNAs) with 3'-end biotin labeling and negative control 3'-biotin-labeled control miRNA sequences were transfected into the cells using RNAiMAX transfection reagent (Thermo Fisher Scientific, Invitrogen™, Cat. no. 13778150) according to the manufacturer's protocol, with a final concentration ranging from 10 to 100 nM. At 48 h post-transfection, cells were collected for lysis. Biotinylated miRNAs and their bound mRNAs were captured using streptavidin-coated magnetic beads (50 μL), with non-specific binding removed through washing. The mRNAs in the pull-down complexes were quantified by qRT-PCR. All qRT-PCR reactions were performed in triplicate. For mRNA detection, GAPDH could be used as an internal reference; for miRNA detection, U6 small nuclear RNA could serve as the internal reference.

### Dual luciferase reporter assay

The online bioinformatics tool TargetScan was utilized to predict the binding site of miR-34a-5p on KRT5, and wild-type (Wt) and mutant (Mut) sequences of this region were designed according to the prediction results. These sequences were inserted into luciferase reporter vectors (pGL3-Promoter, Takara, Dalian, China) to generate Wt-KRT5 and Mut-KRT5 constructs. 293 T cells were then co-transfected with either Wt-KRT5 or Mut-KRT5, along with a miR-34a-5p mimic or non-targeting negative control miRNA (miR-NC). Forty-eight hours after transfection, we utilized a dual-luciferase reporter assay kit (Promega, Madison, USA) to assess the activities of Firefly and Renilla luciferase.

### Bioinformatics analyses

The expression levels of KRT5 and DSP in non-small cell lung cancer were evaluated through bioinformatics platforms. Raw RNA-seq data of lung adenocarcinoma (LUAD) were extracted from The Cancer Genome Atlas (TCGA) via the StarBase v3.0 (starbase.sysu.edu.cn) and TCGA biolinks (v2.25.1) interfaces. Differential expression between tumor and adjacent normal tissues was determined using a linear regression model adjusted for batch effects. Matching analysis was performed using GEPIA (http://gepia.cancer-pku.cn/) based on TCGA and Genotype-Tissue Expression (GTEx) datasets. Expression levels were compared using two-tailed Student's t-test, with statistical significance set at *P* < 0.05.

Protein–protein interaction prediction analysis between KRT5 and DSP was conducted using the STRING database (version 12.0, https://string-db.org/). The analysis species was set as Homo sapiens, with KRT5 and DSP input into the system as target proteins. The confidence score of interactions is comprehensively calculated based on multiple sources of evidence including experimental validation, database annotation, co-expression analysis, and text mining.

### Statistical analysis

Statistical analysis and graphing were performed using GraphPad Prism 9.0 software (GraphPad Software Inc.). The Shapiro–Wilk test was used to assess the normality of measurement data. For comparisons between two groups with normal distribution, unpaired t-tests were employed. For comparisons involving three or more normally distributed groups, one-way ANOVA was selected. Non-normally distributed data were analyzed using rank-sum tests. A P-value < 0.05 was considered statistically significant, and data were presented as Mean ± SD.

## Results

### Downregulated miR-34a-5p and upregulated KRT5 and DSP levels in NSCLC tissues and cells

**Fig. 1:**
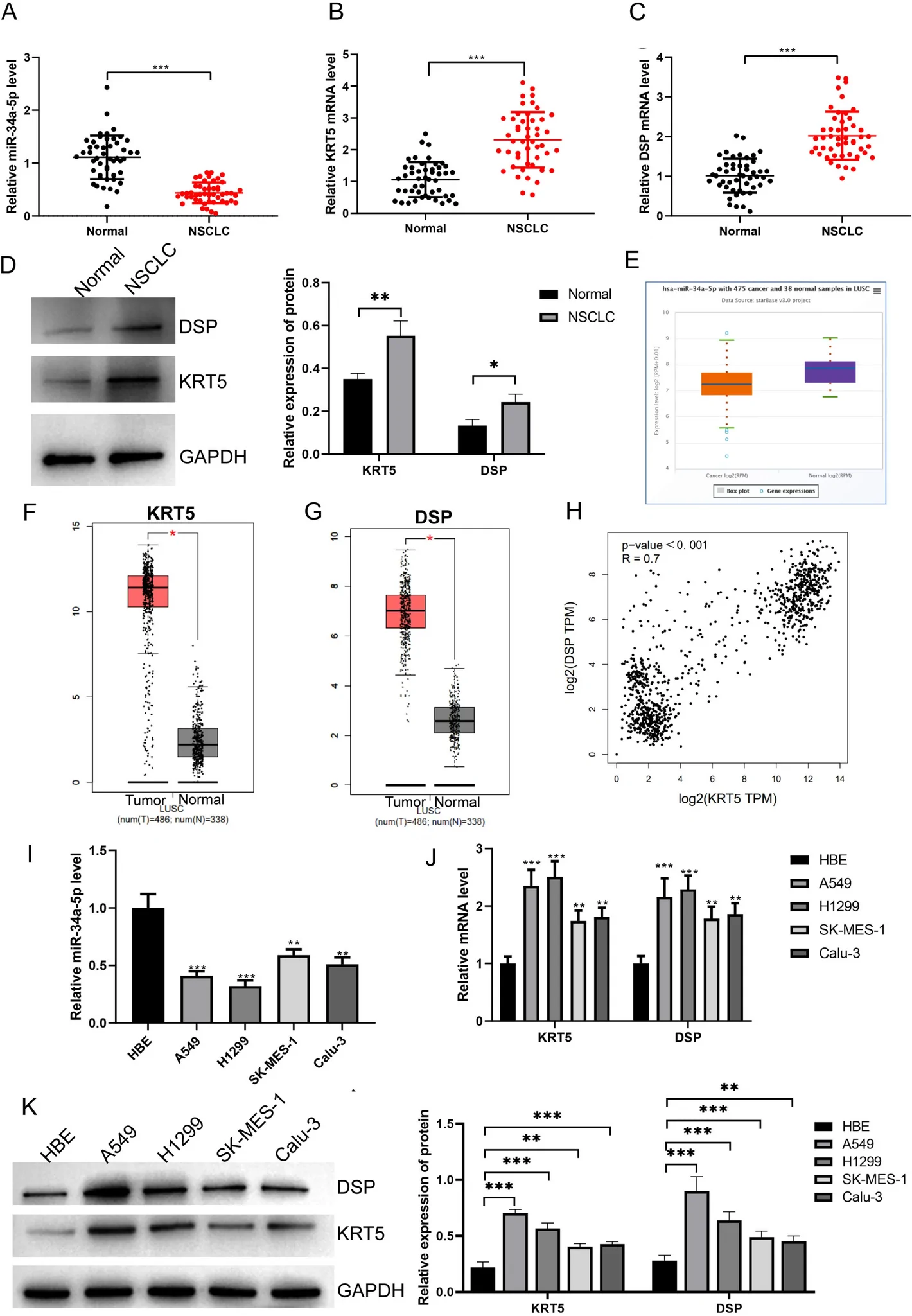
miR-34a-5p was decreasingly expressed and KRT5 and DSP were increasingly expressed in NSCLC tissues and cells. **A–C** RT-qPCR was used to detect the RNA expression levels of miR-34a-5p, KRT5 and DSP in the collected NSCLC and match adjacent normal tissues; **D** Western blotting analysis KRT5 and DSP protein expression in NSCLC and normal tissues; **E–G** StarBase (**E**), TCGA (**F**) and GEPIA (**G**) databases were applied to analyze miR-34a-5p, KRT5 and DSP expression levels in lung cancer tissues and normal tissues; **H** StarBase was used to reveal the correlation between KRT5 and DSP; **I-K** RT-qPCR and western blotting assessed miR-34a-5p, KRT5 and DSP expression levels in NSCLC cell lines, i.e. A549, H1299, SK-MES-1 and Calu-3; n = 3, *p < 0.05, **p < 0.01, ***p < 0.001. NSCLC, non-small cell lung cancer

In this study, the expression levels of specific RNA molecules, namely miR-34a-5p, KRT5, and DSP, were systematically assessed in a cohort of lung cancer samples. A total of 48 NSCLC tissues were collected and analyzed alongside an equal number of matched adjacent normal tissues. This approach allowed for a direct comparison between the tumor tissues and the corresponding normal tissues, providing insights into the differential expression patterns of these RNA markers in the context of NSCLC. The results indicated a significant decrease in miR-34a-5p expression (Fig. 1A) alongside an increase in KRT5 and DSP mRNA levels in NSCLC tissues (Fig. 1B-C). Western blot analysis further confirmed that NSCLC tissues exhibited elevated KTR5 and DSP protein levels compared to normal tissues (Fig. 1D). The data obtained from the StarBase, Cancer Genome Atlas (TCGA), and GEPIA databases supported these findings (Fig. 1E-G). The integration of these datasets strengthens the credibility of the results. The utilization of multiple reputable sources adds depth to the analysis and supports the conclusions drawn from the experimental data.

Furthermore, analysis of the StarBase database identified a positive correlation between keratin 5 (KRT5) expression levels and desmoplakin (DSP) expression in NSCLC tissue samples. (Fig. 1H). We additionally examined correlations between miR-34a-5p and KRT5 mRNA and patients’ clinicopathological characteristics. (Table 1). Our findings revealed significant correlations between the expression of miR-34a-5p and KRT5 mRNA and tumor-node-metastasis (TNM) stage as well as lymph node metastasis, whereas no such associations were observed with patient age, gender, pathological subtype, or smoking history.

Furthermore, we evaluated the expression levels of miR-34a-5p, KRT5, and DSP across four NSCLC cell lines (A549, H1299, SK-MES-1, Calu-3). These analyses confirmed reduced miR-34a-5p expression. (Fig. 1I) In comparison to human bronchial epithelial (HBE) cells, Keratin 5 (KRT5) and Desmoplakin (DSP) were found to be significantly elevated in NSCLC cell lines (Fig. 1 J-K). This observation aligns with the results obtained from clinical samples, further supporting the notion that KRT5 and DSP expression patterns could be essential indicators in the progression of NSCLC. Meanwhile, miR-34a-5p, KRT5 and DSP showed more pronounced alterations in A549 and H1299 cells, leading to their selection for subsequent studies.

### Restoration of miR-34a-5p blunts the CSC phenotype of NSCLC cells

**Fig. 2:**
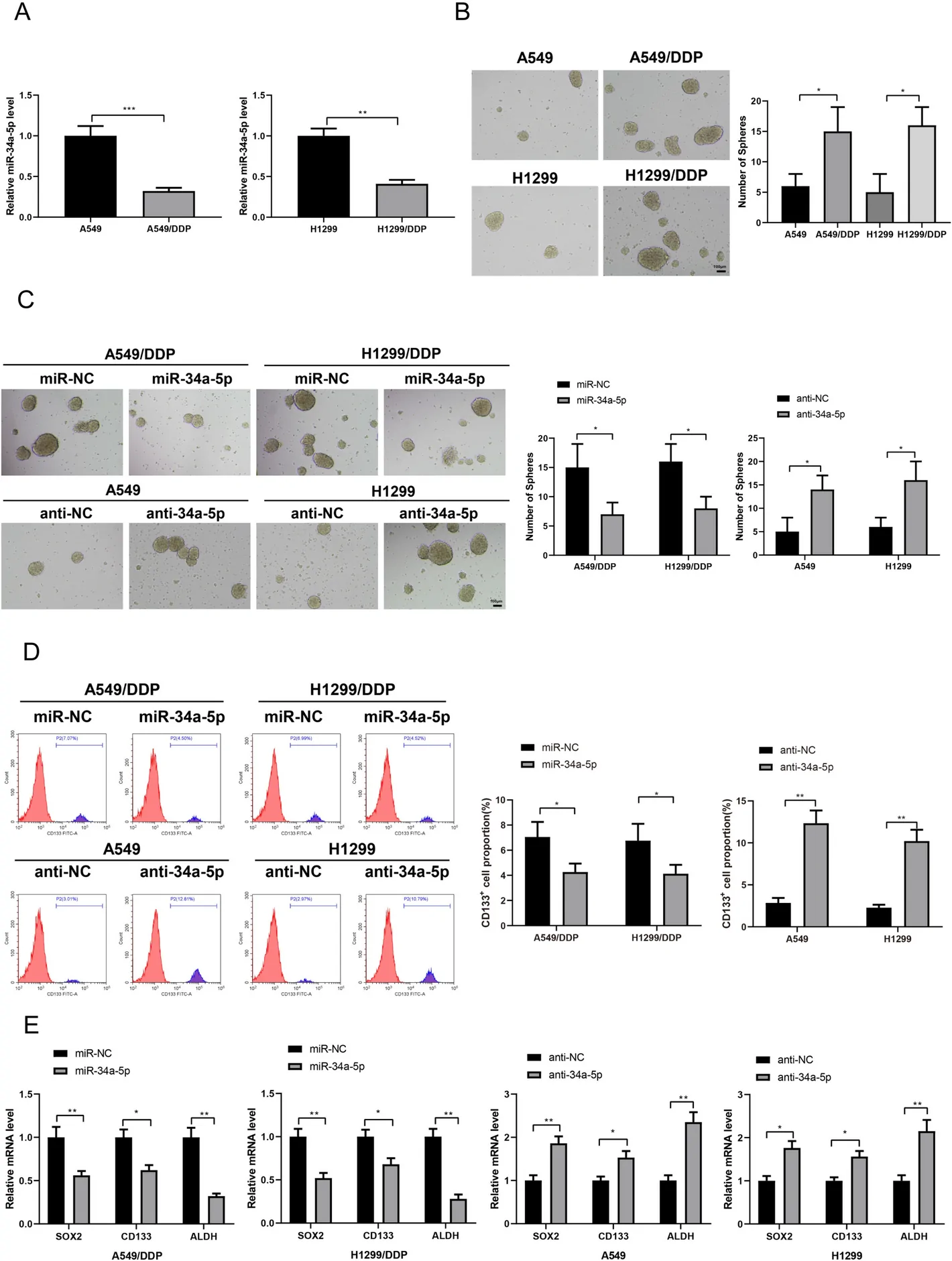
Restoration of miR-34a-5p reduced the CSC properties of NSCLC cells. **A** miR-34a-5p expression in A549/DDP (H1299/DDP) and A549(H1299) cells was detected by RT-qPCR; **B, C** Sphere formation assay was used to assess the number and size of spheres formed by A549/DDP (H1299/DDP) and A549(H1299) cells; **D** CD133 expression in A549/DDP (H1299/DDP) and A549(H1299) cells was evaluated by flow cytometry; **E** RT-qPCR determined the expression of stemness-associated genes (SOX2, CD133, and ALDH); n = 3, *p < 0.05, **p < 0.01, ***p < 0.001. NSCLC, non-small cell lung cancer; DDP, cisplatin; CSC, cancer stem cell

To verify the regulation of CSC-like properties, specifically. chemoresistance, by miR-34a-5p, a sphere formation assay was conducted in NSCLC cells expressing miR-34a-5p. Initially, RT-qPCR analysis demonstrated that A549/DDP and H1299/DDP cells had markedly reduced miR-34a-5p when compared to A549 and H1299 cells (Fig. 2A). In serum-free culture conditions supplemented with growth factors, A549/DDP, H1299/DDP, A549, and H1299 cells exhibited impaired adhesion to the plates and consequently formed a higher quantity of spheres. Importantly, A549/DDP and H1299/DDP cells yielded spheres that were not only larger but also more numerous than those generated by their A549 and H1299 counterparts (Fig. 2B). In A549/DDP (H1299/DDP) cells that overexpressed miR-34a-5p, both the size and the number of spheres diminished; conversely, inhibiting miR-34a-5p in A549 (H1299) cells (anti-miR-34a-5p group) led to an increase in the sphere count and size (Fig. 2C).

Subsequently, the expression of CD133, a marker of lung CSCs, in the spheres was assessed to identify the CSC phenotype. The presence of CD133 + cells suggests self-renewal and tumorigenesis. A549/DDP(H1299/DDP) spheres expressed higher CD133 expression than A549(H1299) spheres; miR-34a-5p overexpression decreased the percentage of CD133 + cells, whereas its inhibition elevated CD133 + cell abundance. (Fig. 2D). Furthermore, RT-qPCR results demonstrated that stemness-associated genes, including SOX2, CD133, and aldehyde dehydrogenase (ALDH), were expressed at lower levels when miR-34a-5p was overexpressed in A549/DDP(H1299/DDP) cells, while inhibition of miR-34a-5p expression in A549(H1299) cells led to an upregulation of these genes (Fig. 2E). These results verify that miR-34a-5p impairs the stemness of NSCLC cells with stem-like properties.

### miR-34a-5p upregulation represses DDP resistance in NSCLC cells

**Fig. 3:**
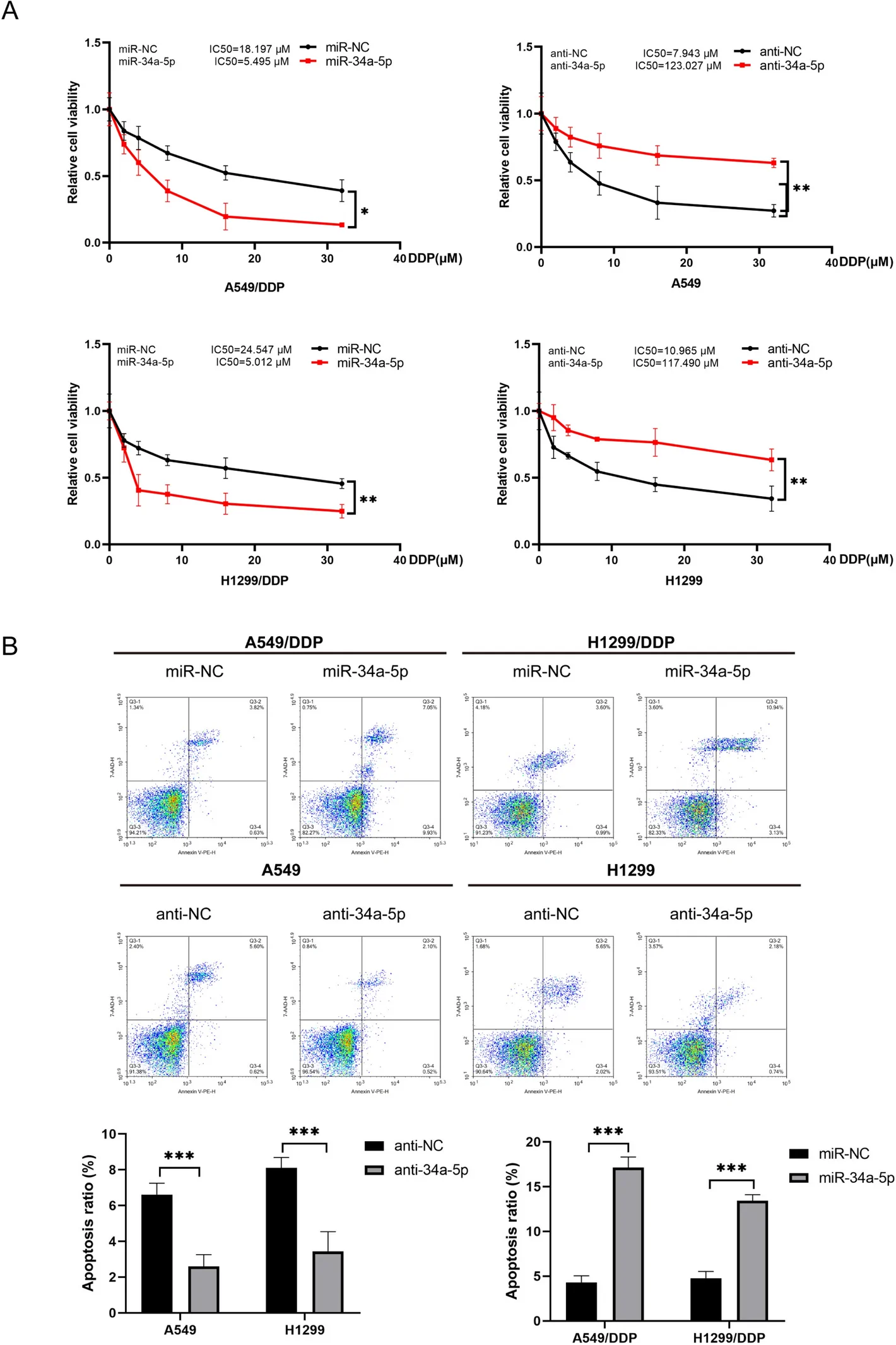
Restoration of miR-34a-5p suppressed the resistance of NSCLC cells to DDP. **A** CCK-8 assay was used to evaluate the resistance of cells to DDP; **B** The apoptosis rate of NSCLC cells were determined by Annexin-PE/7-AAD staining; n = 3, *p < 0.05, **p < 0.01. NSCLC, non-small cell lung cancer; DDP, cisplatin

CSCs exhibit inherent resistance to chemotherapy, which facilitates their survival in the presence of anti-tumor drugs and supports the regeneration of other cancer cell populations. Consequently, the CCK-8 assay was employed to assess the effect of miR-34a-5p on the resistance of sphere-forming cells to DDP. As anticipated, overexpression of miR34-a-5p rendered A549/DDP(H1299/DDP) cells more sensitive to DDP treatment, while A549(H1299) cells with downregulated miR-34a-5p demonstrated increased resistance to DDP (Fig. 3A). Subsequently, the apoptosis rate of the cells was assessed through the use of Annexin-PE/7-AAD staining. The findings revealed that there was an increased proportion of apoptotic A549/DDP (H1299/DDP) cells when miR-34a-5p was overexpressed. This observation suggests that the overexpression of miR-34a-5p significantly improves the sensitivity of A549/DDP (H1299/DDP) cells to the chemotherapeutic agent DDP. In contrast, when miR-34a-5p was downregulated, there was a noticeable reduction in the apoptosis rate of A549 (H1299) cells, indicating a different response to DDP treatment in the absence of this microRNA (Fig. 3B). Overall, these results collectively suggest that miR-34a-5p plays a crucial role in enhancing the sensitivity of stem-like non-small cell lung cancer (NSCLC) cells to DDP and also promotes increased rates of apoptosis in NSCLC cells.

### miR-34a-5p overexpression targets and inhibits KRT5 in NSCLC cells

**Fig. 4:**
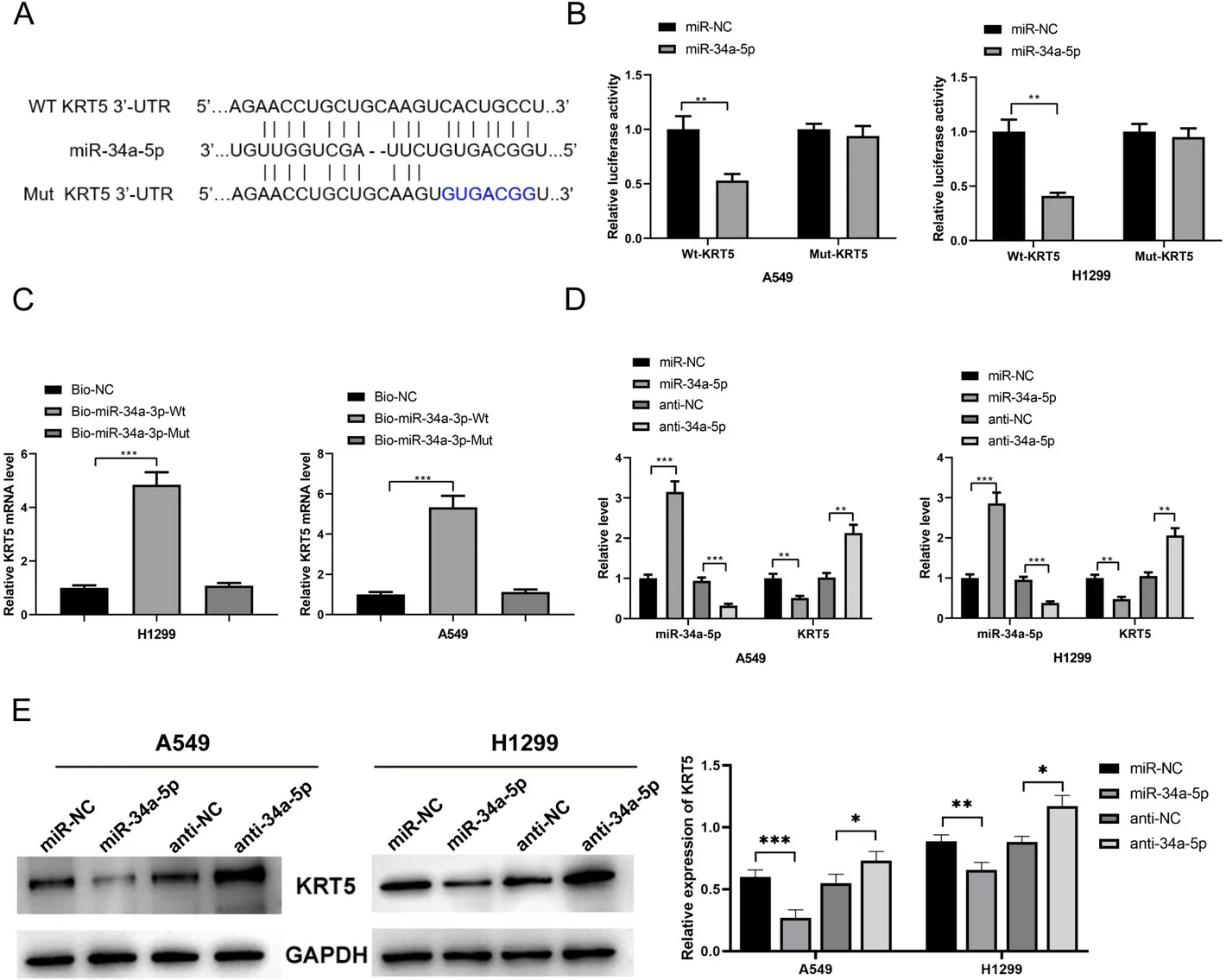
KRT5 was a target gene of miR-34a-5p. **A** Bioinformatics online software StarBase (http://starbase.sysu.edu.cn/) predicted a binding site of miR-34a-5p to KRT5 3’UTR; **B** Dual luciferase reporter assay evaluated the binding relationship between miR-34a-5p and KRT5; **C** RNA pull down assay determined the interaction between miR-34a-5p and KRT5; **D, E** RT-qPCR and western blot analyses were utilized to assess miR-34a-5p and KRT5 expression in cells expressing or knocking down miR-34a-5p; n = 3, *p < 0.05, **p < 0.01, ***p < 0.001

The online tool starBase (http://starbase.sysu.edu.cn/) identified a potential binding site of miR-34a-5p within the 3’UTR of KRT5. (Fig. 4A). Using a dual-luciferase reporter assay, we verified the direct interaction between miR-34a-5p and the KRT5 gene (Fig. 4B). Notably, ectopic expression of miR-34a-5p suppressed luciferase activity in A549 and H1299 cells transfected with the wild-type KRT5 reporter vector (Wt-KRT5), whereas this inhibitory effect was absent in cells transfected with the mutant KRT5 construct (Mut-KRT5). Additionally, KRT5 probe pull-down assay suggested that the biotinylated wild-type miR-34a-5p probe could enrich KRT5 mRNA in NSCLC cells (Fig. 4C). Furthermore, miR-34a-5p mimics and inhibitors were successfully introduced into NSCLC cells (Fig. 4D), Our results demonstrated that overexpression markedly downregulated KRT5 mRNA and protein levels, while its inhibition significantly upregulated these two molecular expressions (Fig. 4D-E).

### KRT5 binds to DSP and promotes DSP expression

**Fig. 5:**
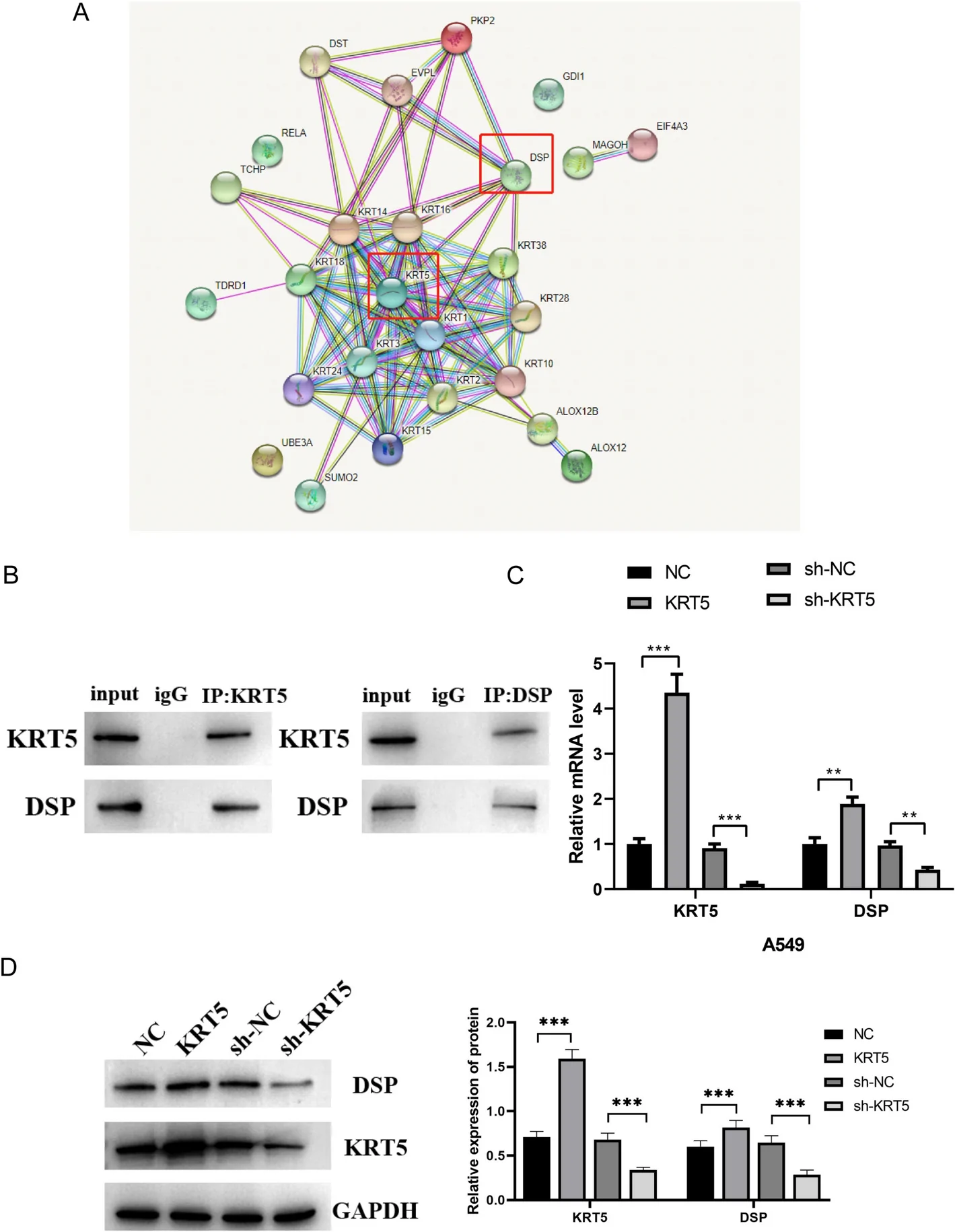
KRT5 bound to and regulate DSP. **A** STRING website suggested the interaction between KRT5 and DSP; **B** Co-Immunoprecipitation identified the interplay of KRT5 to DSP; **C, D** RT-qPCR and western blot analyses assessed KRT5 and DSP expression levels in cells with KRT5 overexpression or silencing; n = 3, *p < 0.05, **p < 0.01, ***p < 0.001

The STRING website indicated a significant interaction between KRT5 and DSP (Fig. 5A). Additionally, Co-IP assay further confirmed this interaction (Fig. 5B). Notably, upon overexpression or silencing of KRT5, the mRNA and protein of DSP were correspondingly upregulated or downregulated (Fig. 5C-D). This indicates that KRT5 has a positive effect on the expression of DSP.

### miR-34a-5p suppresses the CSC phenotype of NSCLC cells and resistance to DDP via KRT5/DSP axis

**Fig. 6:**
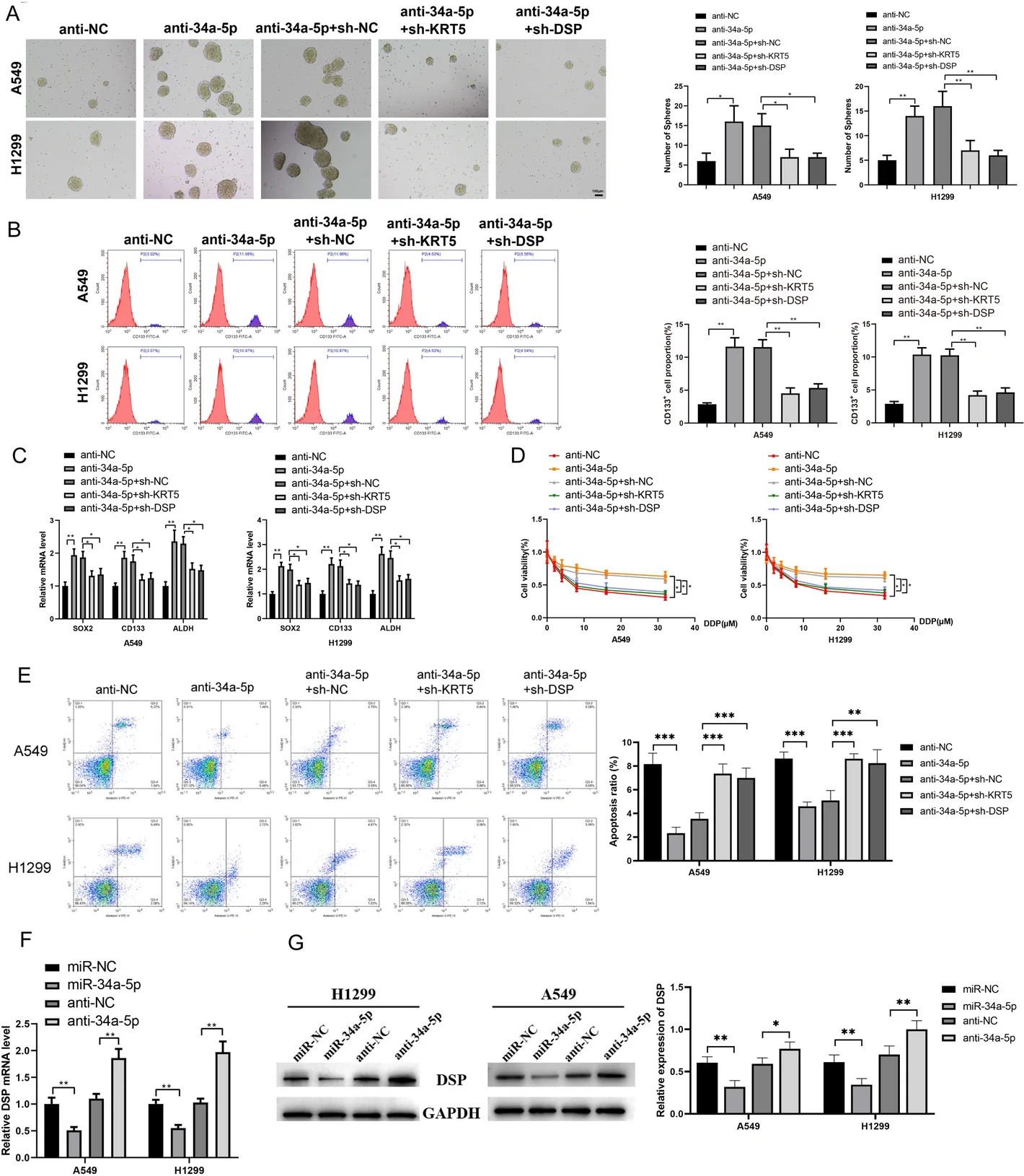
miR-34a-5p regulated KRT5/DSP axis to blunt the stemness of NSCLC cells and resistance to DDP. **A** Number and sizes of spheres formed by A549(H1299) cells; **B** CD133 expression was analyzed by flow cytometry; **C** RT-qPCR assay was utilized to measure the expression levels of SOX2, CD133, and ALDH; **D** Resistance of the cells to DDP was evaluated using CCK-8 assay; **E** Annexin-FITC/PI staining was used to detect the apoptosis rate; **F, G** RT-qPCR and western blotting assessed DSP expression in cells overexpressing or knocking down miR-34a-5p; n = 3, *p < 0.05, **p < 0.01, ***p < 0.001. NSCLC, non-small cell lung cancer; DDP, cisplatin

The role of KRT5 in the inhibition of the CSC phenotype by miR-34a-5p was investigated. Silencing KRT5 or DSP in A549(H1299) cells with downregulated miR-34a-5p resulted in a decrease in the number of spheres and an increase in sensitivity to DDP (Fig. 6A). Flow cytometry analysis indicated a reduction in CD133 + expression (Fig. 6B), while RT-qPCR assays demonstrated reduced SOX2, CD133, and ALDH levels in A549 and H1299 cells following KRT5 or DSP silencing—even with miR-34a-5p inhibition (Fig. 6C). Notably, the cisplatin (DDP) resistance triggered by miR-34a-5p inhibition was partially abrogated via KRT5 or DSP downregulation. (Fig. 6D-E). KRT5 and DSP exhibit close structural and functional collaboration with positive regulatory effects. Furthermore, miR-34a-5p overexpression was found to suppress the expression of both KRT5 and DSP, while inhibition of miR-34a-5p significantly increased DSP expression (Fig. 6F-G). Collectively, the KRT5/DSP axis is pivotal in mediating miR-34a-5p-regulated CSC properties in NSCLC cells.

### miR-34a-5p restrains CSC properties in NSCLC tumors

**Fig. 7:**
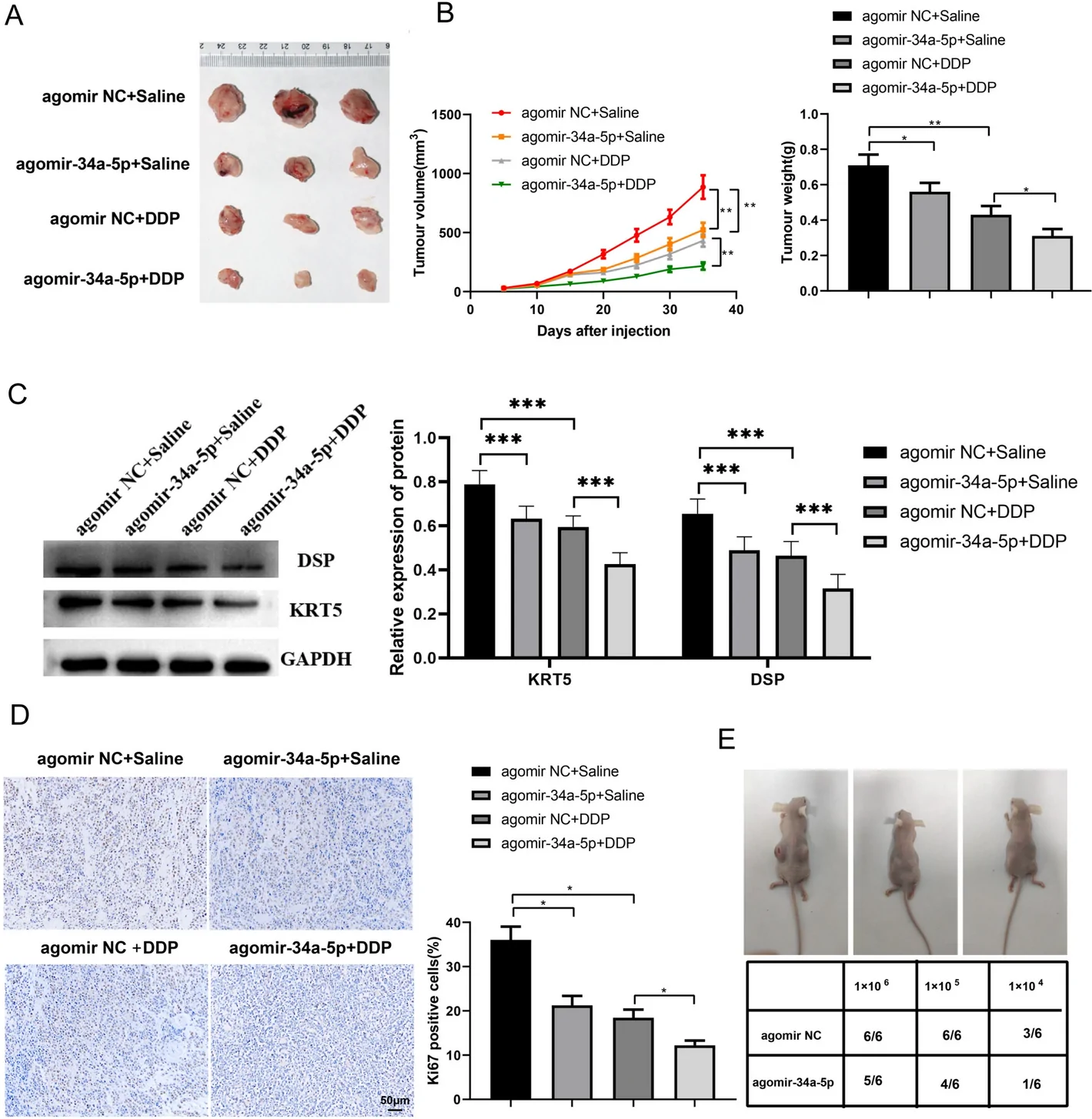
miR-34a-5p regulated CSC properties of NSCLC cells in vivo. **A** Tumors of mice in xenograft assays; **B** Comparison of tumor weight and volume; **C** KRT5 and DSP expression levels in tumors were evaluated by western blotting; **D **The results of immunochemistry of Ki67; **E** Mice were injected with A549/DDP cells at indicated concentrations to observe the growth rate of tumors; n = 6, *p < 0.05, **p < 0.01. NSCLC, non-small cell lung cancer; DDP, cisplatin; CSC, cancer stem cell

In xenograft mouse models, A549/DDP cells that were stably transfected with either agomir-34a-5p or agomir-NC were subcutaneously injected into the right abdomen of the mice. This experimental design allows for the evaluation of the biological effects of the agomirs on tumor growth and progression in vivo, providing insights into potential therapeutic applications. The mice were subsequently subjected to either DDP treatment or normal saline injection (control) (Fig. 7A-B). Our findings indicated that the overexpression of miR-34a-5p enhanced the anti-tumor effectiveness of cisplatin (DDP) in vivo, which was evidenced by a marked reduction in both tumor volume and weight. Notably, miR-34a-5p upregulation in mice led to the downregulation of KRT5 and DSP in tumor tissues. (Fig. 7C). Immunohistochemical analysis of Ki67, a marker for cell division, in xenograft tumors suggested that the injection of cells expressing miR-34a-5p increased sensitivity to DDP and reduced cell division (Fig. 7D). To examine how miR-34a-5p influences cancer stem cells (CSCs), A549/DDP cells were administered to the mice in different concentrations. Upon injection of A549/DDP cells at the same concentration, tumor growth in mice injected with cells overexpressing miR-34a-5p was inhibited compared to those receiving agomir NC (Fig. 7E). The results of the xenograft assay in mice corroborated the finding that miR-34a-5p enhanced the anti-tumor effect of DDP and reduced cell proliferation. However, direct characterization of CSC-related markers in xenograft tissues was not performed in this study. Therefore, the conclusion that miR-34a-5p suppresses CSC properties in vivo remains to be further validated. Nevertheless, the in vivo results are consistent with the in vitro observations that miR-34a-5p reduces DDP resistance.

## Discussion

This study systematically investigated the role of the miR-34a-5p/KRT5/DSP axis in cancer stem cell (CSC) characteristics and cisplatin resistance in NSCLC. Our results demonstrated that miR-34a-5p was significantly downregulated in NSCLC tissues and cells, while KRT5 and DSP were markedly upregulated. Functional experiments further confirmed that miR-34a-5p could attenuate the stem-like phenotype of NSCLC cells and enhance their sensitivity to DDP by targeting and suppressing KRT5, thereby subsequently affecting DSP expression. These findings suggest that miR-34a-5p is not only one of the molecular events associated with NSCLC progression, but may also serve as a crucial upstream regulatory factor linking cancer stemness maintenance with chemotherapy resistance.

Previous studies have demonstrated that the relevant miR-34a is downregulated in LUAD and associated with EMT and CSC characteristics in lung cancer[19]. The miR-34 family is widely recognized for its tumor-suppressive effects[20, 21]. It is involved in regulating various biological processes such as cell proliferation, apoptosis, epithelial-mesenchymal transition, stemness maintenance, and therapeutic response.

Previous studies have shown that abnormal downregulation of miR-34a is associated with poor progression in various malignant tumors, and its restored expression can partially reverse the malignant phenotype of tumor cells[22]. This study further focuses the role of miR-34a-5p on CSC properties and cisplatin resistance in NSCLC, demonstrating that the loss of miR-34a-5p may not only promote tumor cell growth but also contribute to treatment failure by enhancing the "stem-like" characteristics of tumor cells. Since the CSC subpopulation typically possesses stronger self-renewal capacity, anti-apoptotic ability, and drug efflux capability, our findings mechanistically support the pathological cascade of "miRNA imbalance—CSC enrichment—enhanced drug resistance".

It is noteworthy that this study identified KRT5 as a direct target gene of miR-34a-5p and suggested DSP as a key effector molecule in this regulatory network. As a basal-like epithelial marker, the biological significance of KRT5 is not entirely consistent across different lung cancer subtypes. However, growing evidence suggests that its aberrant expression may be associated with enhanced tumor aggressiveness, altered cell lineage plasticity, and therapy resistance. Previous studies in breast tumors have indicated that KRT5 is positively correlated with the CSC marker CD44, and is associated with progenitor-like characteristics and enhanced chemotherapy resistance[23, 24]. This study observed similar phenomena in NSCLC, suggesting that KRT5 may not only serve as a phenotypic marker but also directly participate in maintaining the CSC state. Furthermore, we found that KRT5 is positively correlated with DSP and can promote DSP expression, indicating that the KRT5/DSP axis may be involved in maintaining tumor cell structural stability, intercellular adhesion status, and stress adaptation capacity, thereby influencing cellular tolerance to chemotherapeutic drugs. Although the deeper molecular connections between them require further elucidation, this study provides new evidence for understanding the synergistic role of KRT5 and desmosome-related molecules in NSCLC stemness and drug resistance.

As previously reported, DSP was strongly expressed in LSCC [16]. But in LUAD, miR-34b/c was reported to suppress cancer development through enhancing DSP expression [18]. This study found that DSP is upregulated in NSCLC tissues and cells, positively correlated with KRT5 expression, and jointly participates in the regulation of CSC phenotype and DDP resistance through the miR-34a-5p/KRT5 axis. These results suggest that DSP may play a subtype-dependent or context-dependent role in NSCLC. The observed differences may be attributed to variations in sample composition, histological subtypes of lung cancer, tumor microenvironment status, and distinct signaling networks involved.

The findings of this study possess certain translational potential. The downregulation of miR-34a-5p and upregulation of KRT5/DSP were associated with TNM stage and lymph node metastasis, suggesting this axis may participate in NSCLC invasion. Cisplatin resistance remains a therapeutic bottleneck in NSCLC treatment. Restoring miR-34a-5p expression or blocking the KRT5/DSP axis could theoretically enhance chemosensitivity, particularly in cancer stem cell-enriched populations and drug-resistant subgroups. While these hypotheses require further validation, the current study provides experimental evidence for developing relevant precision therapeutic strategies. This study also has several limitations. First, the sample size was limited and derived from a single center, and the generalizability and statistical stability require validation in larger, multicenter cohorts. Second, the in vitro experiments were conducted only with A549 and H1299 cells, which may not fully capture the molecular heterogeneity of NSCLC. The role of this axis across different histological subtypes and genetic backgrounds remains to be confirmed. Finally, although this study demonstrated that miR-34a-5p directly targets KRT5 and the functional association between KRT5 and DSP, the specific mechanism by which KRT5 regulates DSP (e.g., transcriptional regulation, protein stability, or signaling pathways) remains unclear and warrants further investigation.

In summary, this study reveals the crucial tumor-suppressive role of miR-34a-5p in NSCLC, demonstrating that it can attenuate CSC properties and enhance tumor cell sensitivity to cisplatin by targeting and inhibiting the KRT5/DSP axis. This finding not only deepens our understanding of the molecular basis underlying stemness maintenance and chemoresistance in NSCLC, but also suggests that the miR-34a-5p/KRT5/DSP axis may serve as a novel therapeutic target for future interventions against drug resistance and tumor recurrence. Further large-scale clinical validation and in-depth mechanistic studies are still required to facilitate the clinical translation of this pathway.

## Supplementary Information

Additional file1 (DOCX 590 KB)

## Data Availability

The datasets generated and/or analysed during the current study are available in the TCGA repository, https://portal.gdc.cancer.gov/ (accession: TCGA-LUAD), Ualcan，https://ualcan.path.uab.edu/analysis-mir.html and the GTEx repository, https://gtexportal.org/home/ (accession: GTEx v8). The protein‑protein interaction data were retrieved from the STRING database (version 12.0, https://string-db.org/).
