# Supplementary material for: miR-34a-5p-mediated inhibition on the KRT5/DSP axis blunts cancer stem cell properties and cisplatin resistance in non-small cell lung cancer
Source: Discov Oncol. 2026 Jun 23;17:1301. doi: 10.1007/s12672-026-05424-z (PMC13547569; doi:10.1007/s12672-026-05424-z)
Supplement: Supplementary file 1 [file 12672_2026_5424_MOESM1_ESM.docx]

Fig 1. Validation of transfection efficiency in each group of cells.

Fig 2. The IC50 assay of cisplatin confirmed that A549/DDP and H1299/DDP are drug-resistant cell lines.
